# Proportioning Factors of Alkali-Activated Materials and Interaction Relationship Revealed by Response Surface Modeling

**DOI:** 10.3390/ma16052042

**Published:** 2023-03-01

**Authors:** Jun Zhao, Aiguo Wang, Bangcheng Lyu, Kaiwei Liu, Yingjie Chu, Rui Ma, Haiyan Xu, Yan Jing, Daosheng Sun

**Affiliations:** 1 Anhui Province Engineering Laboratory of Advanced Building Materials, Anhui Jianzhu University, Hefei 230601, China; 2 School of Materials Science & Engineering, Southeast University, Nanjing 211189, China

**Keywords:** alkali-activated material, response surface model, curing condition, interaction

## Abstract

Alkali-activated fly-ash–slag blending materials (AA-FASMs) are gradually being studied and applied more because of their good performance. There are many factors affecting the alkali-activated system, and the effect of single-factor variation on the performance of AA-FASM has been mostly reported; however, there is a lack of unified understanding of the mechanical properties and microstructure of AA-FASM under curing conditions and multiple-factor interaction. Therefore, this study investigated the compressive strength development and reaction products of alkali-activated AA-FASM under three curing conditions including seal (S), dry (D) and water saturation (W). Based on the response surface model, the relationship between the interaction of slag content (*W_SG_*), activator modulus (*M*) and activator dosage (*R_A_*) on its strength was established. The results showed that the maximum compressive strength of AA-FASM after 28 days of sealed curing was about 59 MPa, while the strengths of dry- and water-saturation-cured specimens decreased by 9.8% and 13.7%, respectively. The seal-cured samples also had the smallest mass change rate and linear shrinkage and the most compact pore structure. Due to the adverse effects from a too-high or too-low modulus and dosage of the activators, the shapes of upward convex, slope and inclined convex were under the interaction of *W_SG_*/*M*, *W_SG_*/*R_A_* and *M*/*R_A_*, respectively. The correlation coefficient *R*^2^ > 0.95 and *p*-value < 0.05 indicated that the proposed model could be used to predict strength development given the complex factors. Optimal proportioning and curing conditions were found to be *W_SG_* = 50%, *M* = 1.4, *R_A_* = 50% and sealed curing.

## 1. Introduction

The CO_2_ emissions generated from cement production account for 5–7% of the total CO_2_ emissions in the world [1]. With the increasing demand for cement in developing countries, the total CO_2_ emissions are still rising, which has become one of the major factors initiating the global temperature rise and climate warming [2,3]. The research on modern building materials has focused on the development of green cementitious materials with a low carbon footprint. Compared with ordinary Portland cement (OPC), the manufacture of alkali-activated cement products can reduce carbon emissions by 26~45%, which has been considered a potential alternative source of OPC [4].

The main raw materials for the manufacture of alkali-activated cement include aluminum-rich silicate materials such as metakaolin and fly ash, which are dominated by geopolymerization, and calcium-rich materials such as slag [5]. Low-polymerization-degree silicate ion ([SiO(OH)_3_]^−^) and aluminate ion ([Al(OH)_4_]^−^) undergo a polycondensation reaction under alkaline conditions, and the reaction product formed in alkali-activated fly ash (FA) is an aluminosilicate-type gel with a three-dimensional network structure [6]. Meanwhile, C-(A)-S-H gel with Ca/Si ratios (0.8−1.1) is usually obtained from the alkali-activated slag [7,8].

In recent decades, raw materials for manufacturing alkali-activated cement have been changed from natural minerals to industrial by-products [9,10], such as alkali-activated fly-ash–slag materials (AA-FASMs). The performances of AA-FASM are significantly affected by the proportion of the binder, modulus and dosage of the activator, curing conditions and other factors [11,12,13,14,15,16]. For instance, Nath et al. [12] showed that the setting time and strength were significantly reduced due to the increased slag content in AA-FASM. Lee et al. [17] pointed out that the increase in the modulus (*M*) of water glass (Na_2_O·nSiO_2_) from 0.5 to 1.0 decreased the compressive strength by 28.9%, which was due to the reduction in the alkalinity of the alkali activator. Moreover, Oderji et al. [18] investigated the effect of solid sodium silicate activator dosage on the geopolymer microstructure under a constant fly ash/slag ratio and observed that the microstructure became more homogeneous with the increase in sodium silicate dosage (mass ratio) from 7% to 8%.

Curing conditions also have an important impact on the performance and microstructure of cementitious materials [16,19]. Fluctuation in curing humidity will cause a change in water content in the paste. Huang et al. [20] demonstrated that a sealed-curing condition could mitigate the leaching of OH^−^ and other ions from the alkali-activated materials and thus maintain high alkalinity in the matrix. Mastali et al. [21] observed that under low humidity, drying shrinkage was intensified due to the evaporation of a large amount of unbound water that existed in alkali-activated materials. Compared with OPC, the shrinkage dynamics of alkali-activated slag (AAS) are more easily affected by the change in relative humidity (RH) and will exhibit the characteristics of hysteresis shrinkage [22]. In addition, there is also a strong relationship between efflorescence and humidity [23]. The reaction process of efflorescence is shown in the following formulas:

CO_2_(g) + 2OH^−^(aq)→CO_3_^2−^(aq) + H_2_O

2Na^+^(aq) + CO_3_^2−^ + 7H_2_O→Na_2_CO_3_·7H_2_O(s)

Although alkali-activated materials have excellent mechanical strength and durability [11], the common “single-factor” research cannot reflect the potential interaction between various factors. The setting time, strength and other properties of AA-FASM cannot be predicted easily. Therefore, it is important to study the influence of these factors and curing conditions on the properties of AA-FASM.

In this paper, the effects of curing conditions on the basic mechanical properties and product microstructure of AA-FASM were systematically studied. On this basis, the response surface model was introduced into the mix design. The response surface models for the compressive strength of AA-FASM under the condition of interaction factors were also established, and the mechanism behind this model was further discussed.

## 2. Materials and Methods

### 2.1. Materials

**Figure 1:**
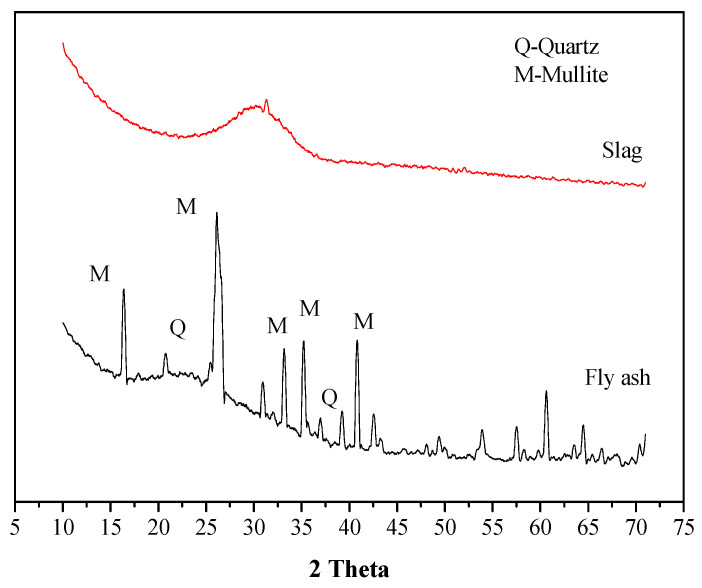
XRD of raw materials.

Industrial solid waste can replace Portland cement as a cementitious material. The spherical particle fly ash produced in the coal-burning process of thermal power plants mainly contains aluminosilicate components and has an annual output of 900 million tons [2]. Slag is produced by smelting pig iron and is a potentially active mineral material. At present, although slag is only partially used in the production of cement and concrete, it can also achieve green energy saving and low carbon in the construction industry [13]. A large amount of industrial solid waste needs to be stored and treated to avoid serious environmental problems. Alkali-activated material is a new form of solid-waste utilization, and fly ash and slag activated by alkaline solution are beneficial to improve the utilization rate of solid waste. The mixture of fly ash and slag may solve the problems of low activity of fly ash and rapid set of alkali-activated slag and will further expand its engineering application prospects [17]. The chemical composition and loss on ignition of fly ash and slag used in the experiment are shown in Table 1. Figure 1 shows the mineral composition of fly ash and slag, which have distinct quartz and mullite crystals in fly ash and an amorphous phase with a hump mainly between 20 and 35° in slag. Fly ash has a high percentage of SiO_2_ and Al_2_O_3_ oxides, while slag has a calcium oxide percentage of 38.25%. Commercially available sodium silicate solution was used in this study, with the composition of 32.35% SiO_2_, 13.73% Na_2_O and 53.92% water, which has a 2.43 mass ratio of SiO_2_ to Na_2_O. Required alkali activator (constant solid content of 40%, different activator modulus (1.0, 1.4 and 1.8)) was prepared by mixing the flake sodium hydroxide solid (≥99% purity), water and sodium silicate solution, which was then cooled to room temperature (>4 h) before using.

### 2.2. Experimental Design

AA-FASM was manufactured with constant activator modulus, different slag content and activator dosage under the three curing conditions. Meanwhile, the proportions of slag (*W_SG_* = m (slag)/m (slag + fly ash)), the modulus of alkali activator (*M* = n (SiO_2_)/n (Na_2_O)) and the ratio of alkali activator to binding material (*R_A_* = m (alkali activator)/m (slag + fly ash)) were selected as factors in the establishment of response surface model, and their corresponding levels and design scheme are shown in Table 2 and Table 3, respectively. In order to simplify and unify, the mixing ratio numbers under the three curing conditions adopted were the same as those in the surface model, namely B5, B6, B7, B8 and B13 in Table 3.

The response surface model was established to verify the interaction on the basis of exploring the effects of seal-, dry- and water-saturation-curing conditions on the mechanical properties and microstructure of AA-FASM. Response surface method (RSM) is a conditional optimization method, which is suitable for solving nonlinear data. After the factors and levels were determined, the mathematical model between response value and variables was established according to the optimization of the experimental design and the analysis of the quadratic regression equation. As a result, the optimal parameters could be obtained through the regression equation and analysis [24]. In this paper, Box–Behnken design was used as the experimental design method.

### 2.3. Preparation of Specimens

The pre-mixed fly-ash–slag powder was placed in NJ-160A planetary mixer to dry-mix for 1 min to ensure homogeneity, and the alkali activator was then added into the mixer. After the mixture was stirred for 240 s, which was poured into the mold (cylinder with a diameter of 29 mm and a height of 40 mm) four times and was vibrated 50 times on the NLD-3 cement mortar fluidity meter after each pouring, 6 replicates in each group were used for testing compressive strength. The temperature of three curing conditions was controlled at 20 ± 2 °C, and each specimen was wrapped with film for seal curing and tested for strength for 1 day, 3 days and 28 days, respectively. In terms of dry curing, the relative humidity was 55%; for water-saturation condition, the container needed to be sealed with a film when the specimens were cured in water (only tested 28-day compressive strength).

### 2.4. Test Method

#### 2.4.1. Compressive Strength

The “Method of testing cements-Determination of strength” [25] was used to test the compressive strength of specimens. The average value of strength obtained for each group of 6 specimens was used, and the error was controlled within 10%.

#### 2.4.2. Mass Changes

To determine the mass change rate of AA-FASM under different curing conditions, at least 3 specimens in each group were tested, and each specimen was tested at least twice. There, mass average was used. An analytical balance (accurate to four decimal places) was used to weigh the mass at each curing period, and the rate of mass change was calculated according to Equation (1):

(1) $$M_{L}=\frac{m_{i}-m_{0}}{m_{0}}\times 100\%$$

where *M_L_* and *m_i_* are the rate of mass change (%) and mass (g) in each stage, respectively, and *m*_0_ is the initial mass, g.

#### 2.4.3. Linear Shrinkage

The measurement of shrinkage change was carried out in a comparative study under different curing conditions. Each group had at least 3 specimens, and each specimen was tested at least twice. Vernier caliper (accurate to two decimal places) was adopted to measure the linear shrinkage, and then, the linear shrinkage rate was calculated according to Equation (2):

(2) $$L_{S}=\frac{L_{i}-L_{0}}{L_{0}}\times 100\%$$

where *L_s_* and *L_i_* are the rate of linear shrinkage (%) and shrinkage value (mm), respectively, and *L_0_* is the initial length, mm.

#### 2.4.4. XRD Analysis

The mineral composition of alkali-activated materials was determined with D/max-2500 X-ray diffraction (XRD) instrument (Rigaku, Japan). The testing parameters were set as follows: copper target (Cu kα), working voltage 40 kV, working current 100 Ma, 2θ from 5° to 70° and the pace 2.4°/min.

#### 2.4.5. FT-IR Analysis

The reaction process of AA-FASM with different ratios under three curing conditions was analyzed by using Nicolet 6700 FT-IR, and the scanning range was 400~4000 cm^−1^.

#### 2.4.6. Pore Structure

Based on the principle that the signal quantity of NMR is directly proportional to the quantity of liquid water in the specimen and the ratio between the volume of liquid water to the volume of specimen is the porosity, ^1^H NMR test was carried out on the water-saturated specimen obtained by the negative pressure treatment of water-saturated instrument for 24 h. The volume of water-saturated specimen was determined according to the buoyancy method before testing, and the porosity and pore size distribution were obtained with MesoMR23-060V-1 nuclear magnetic resonance detection equipment.

#### 2.4.7. BSE Morphology

The specimen was broken down into the largest side length of approximately 5 mm. After vacuum drying, the sample was sealed with epoxy resin, waiting until the resin hardened. A polished machine was used to obtain a smooth surface. The high-resolution backscatter image of specimen was obtained with a Zeiss GeminiSEM 500 Schottky field emission scanning electron microscope, and the average Ca/Si was calculated by EDS element analysis.

## 3. Results and Discussion

### 3.1. Effects of Curing Conditions on Performance, Product and Microstructure

#### 3.1.1. Compressive Strength

**Figure 2:**
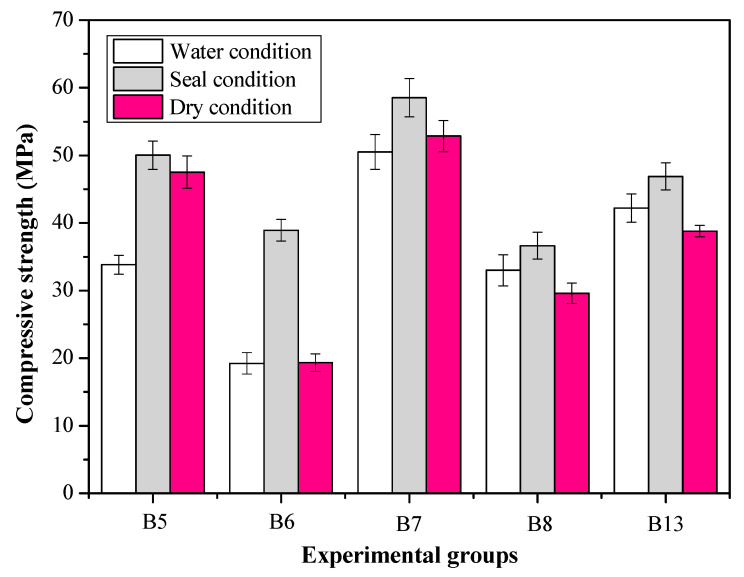
Effect of curing conditions on the 28-day compressive strengths of AA-FASM.

Figure 2 shows the effect of curing conditions on the compressive strengths of AA-FASM cured for 28 days. The results indicate that the water-saturation condition may have a detrimental effect on the geopolymerization of the sample with a high content of fly ash. Although there were no obvious cracks in the samples, the compressive strengths of B5 and B6 decreased by 32.4% and 50.6%, respectively, when compared with the sealed-curing samples. On the contrary, dry curing with low humidity (55% RH) causes internal moisture exchange and microcracks in AA-FASM [26], especially for the compressive strength of B6 (*R_A_* = 55%), which was remarkedly reduced by 50.3%. In addition, S-B7 reached about 59 MPa, while D-B7 and W-B7 decreased, respectively, by 9.8% and 13.7%. Seal curing improves the strength and compactness of the specimens, whether they are single- or high-calcium-component alkali-activated materials [27]. The higher water content is free and can flow within the pore while present in the low-calcium-based system [28], while there may be a lower pH and leaching of the activator during an early water-curing condition [27]. High-humidity environments have side effects on the product structure, weakening the microstructural properties of alkali-activated materials [29,30].

Another change to note is that the strength of the sample with *R_A_* = 55% (B6 or B8) was lower than that of the sample with *R_A_* = 45% (B5 or B7), which may be related to the water content of the system. In the several stages of the reaction of water and alkali-activated material, water can be used as a medium to dissolve the reactant or as a product [31]. Excessive moisture can hinder the improvement of compressive strength. As shown in the following formula, water is precipitated when polycondensation occurs:

(3) $${\mathrm{SiO}(\mathrm{OH})}_{2}+\mathrm{AlOOH}\;\to \;\mathrm{HO}—\underset{O}{\underset{|}{\mathrm{Si}}}—O—\mathrm{Al}+H_{2}O$$

Sealed curing led to the highest compressive strengths of AA-FASM, and the ratio with *W_SG_* < 50% and *R_A_* > 50% was not suitable for curing in a water-saturation and dry environment.

#### 3.1.2. Mass Change Rate

**Figure 3:**
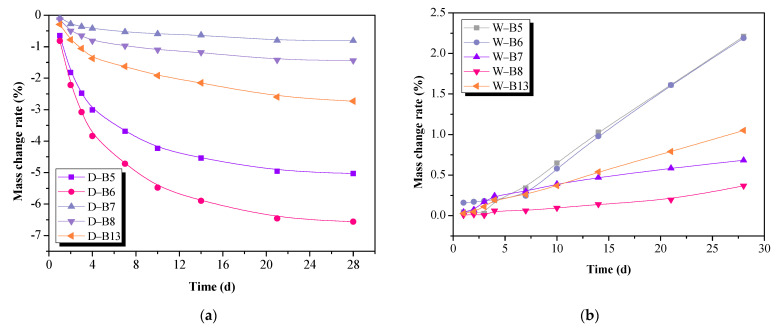

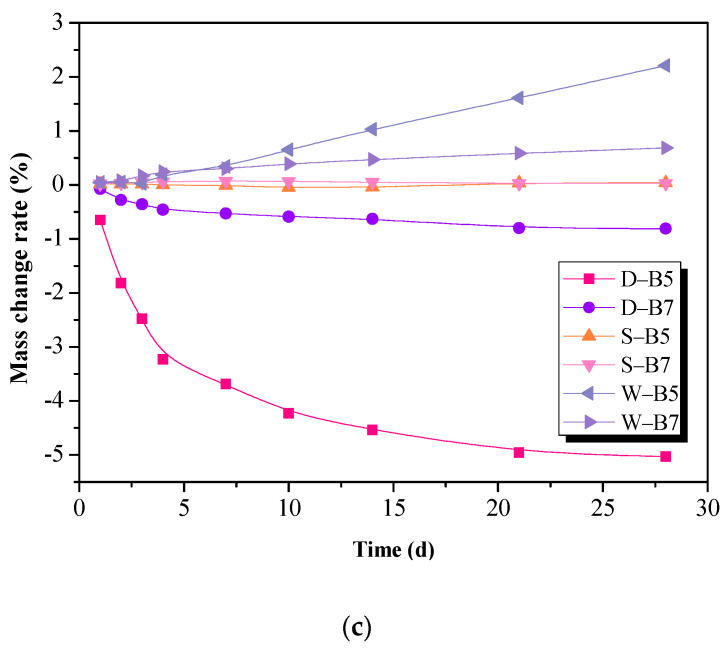
Effect of three curing conditions on the mass change rate of AA-FASM. (**a**) Dry curing. (**b**) Water-saturation curing. (**c**) Mass change rates of B5 and B7.

The effect of three curing conditions on the mass change rates of AA-FASM is shown in Figure 3. The mass changes were directly influenced by the reactivity of raw material and the water content in the matrix. An increase in slag content and a decrease in activator dosage significantly reduced the mass loss of dry-cured samples. The early-age reaction of fly ash was slow, resulting in a large increase in *R_A_* = 45% or 55%, and the decrease in mass change was most prominent with B6 (*W_SG_* = 25%, *R_A_* = 55%), up to 6.6% (Figure 3a). In contrast to dry curing, the mass of water-saturation-cured samples increased with curing age. The samples with a higher content of fly ash (W-B5, W-B6) had larger water absorption (>2.1%), and the mass increase rate of *R_A_* = 55% was lower than *R_A_* = 45% (Figure 3b). In addition, as seen in Figure 3c, the sealing-cured sample maintained a relatively constant mass due to less humidity exchange occurring with the outside. The three curing conditions have their own distinct characteristics and are different from each other. It is worth noting that the loss of water and the increase in humidity will cause greater fluctuations in the mass and strength of AA-FASM.

#### 3.1.3. Linear Shrinkage

**Figure 4:**
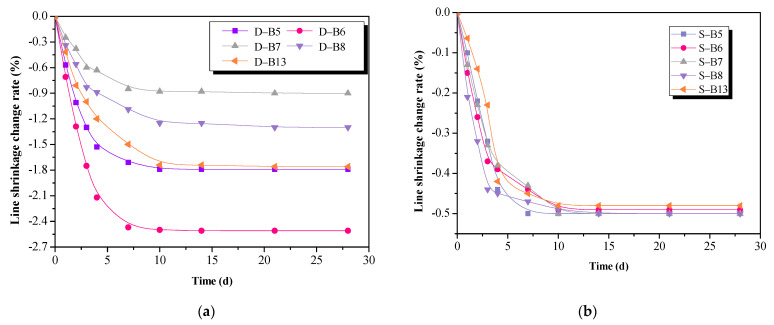

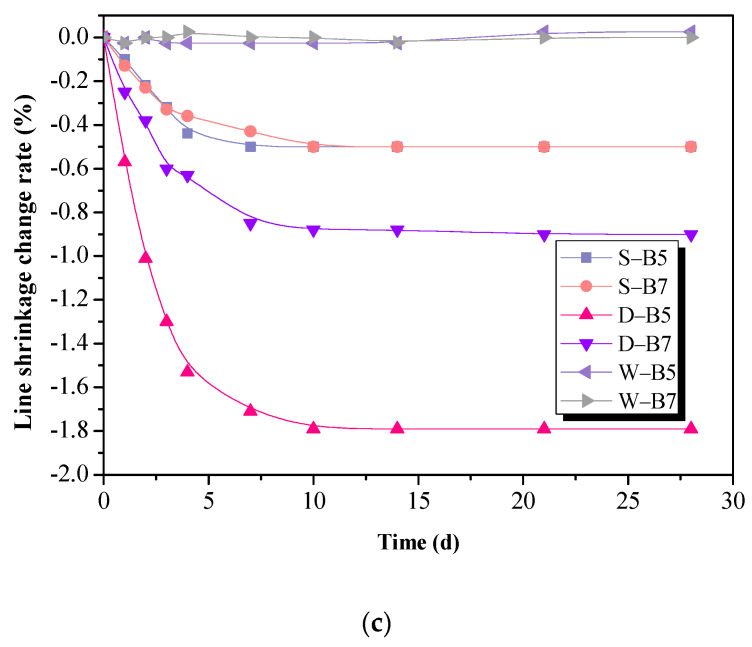
Effect of curing condition on the linear shrinkage of AA-FASM. (**a**) Dry curing. (**b**) Seal curing. (**c**) Linear shrinkage change rate of B5 and B7.

Figure 4 shows the effect of the curing condition on the linear shrinkage of AA-FASM, where negative values represent shrinkage. The linear shrinkage under dry curing corresponds well with the mass change rate. The loss of water increased with the content of capillary pores in the matrix, so the drying shrinkage was larger. High contents of fly ash and the alkali activator are not suitable for the dry-curing samples, such as B6 (*W_SG_* = 25%, *R_A_* = 55%). The linear shrinkage was up to about 2.6%, and the microcracks occurred easily and caused reductions in strength. Sealed curing created a barrier of water exchange with the external drying environment, and only self-shrinkage caused by the chemical reaction was present in AA-FASM, as shown in Figure 4b. Furthermore, the high-humidity condition does not cause the appearance of the capillary meniscus in the paste and thus reduces the possibility of capillary pressure [32], so there was no change in the shrinkage value (see Figure 4c). Combining the changes in mass change rate and linear shrinkage, the loss of water has a greater impact on compressive strength than the surplus of water.

#### 3.1.4. XRD Analysis

**Figure 5:**
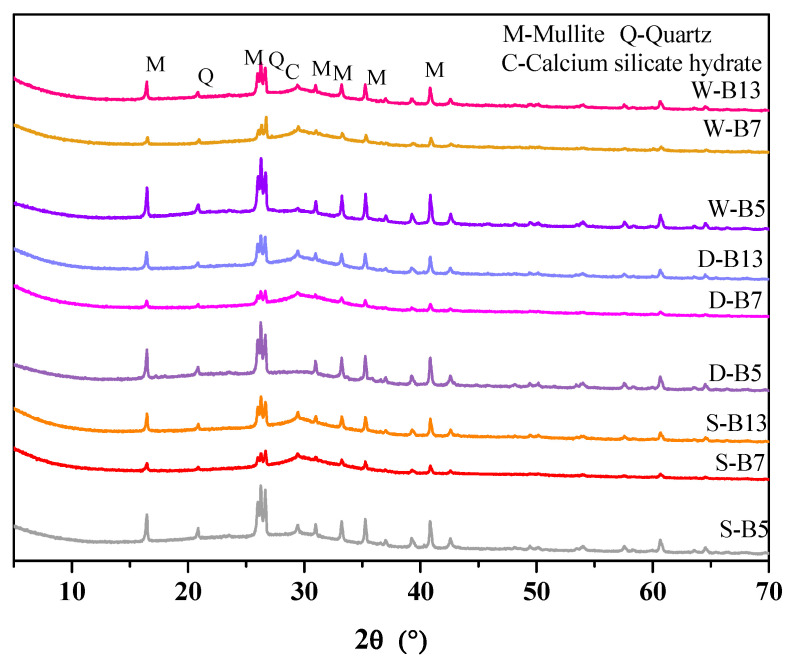
XRD pattern of AA-FASM products under the different curing conditions.

Figure 5 shows the XRD patterns of AA-FASM samples under different curing conditions. There was a wide hump present at 20°~38°, which was associated with the product of N-A-S-H with an amorphous structure. The 2θ at the center of the hum was around 30°, which was the main sign of N-A-S-H gel generation in other studies of fly-ash-based geopolymers [33]. Regardless of the curing condition, the incorporation of slag promoted the formation of a C-A-S-H gel phase. Studies have shown that C-S-H gel can be formed quickly in the early stage of the reaction, which accelerates the dissolution of Si and Al in fly ash and slag, promotes the reaction and accelerates the generation of N-A-S-H gel as a nucleation site, which also makes the matrix more compact [34,35].

#### 3.1.5. FT-IR Analysis

**Figure 6:**
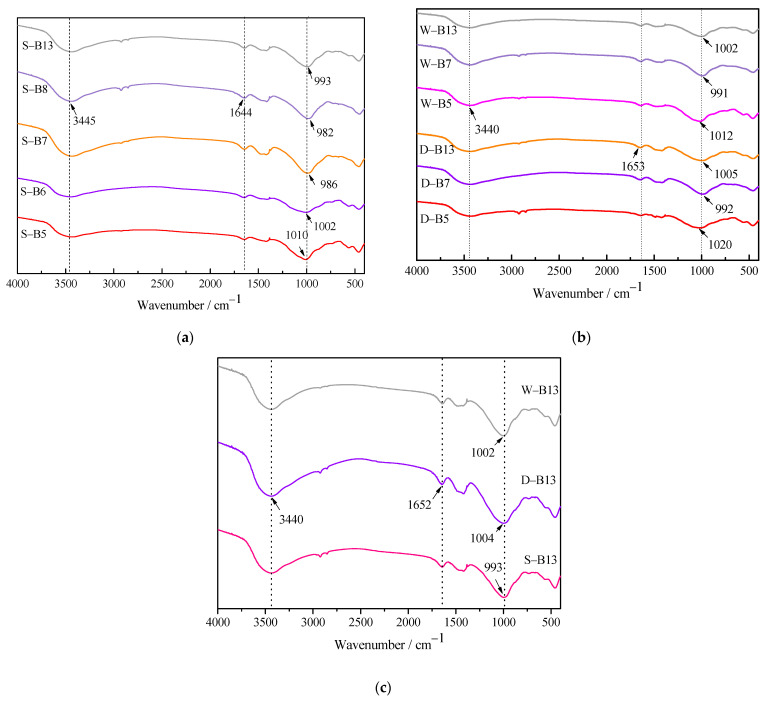
FT-IR spectra of AA-FASM under the different curing conditions. (**a**) Seal curing. (**b**) Dry and water-saturation curing. (**c**) B13.

Figure 6 shows the FT-IR spectrum of AA-FASM under different curing conditions. The peak of the absorption band of the product shifted to a low wave number with the increase in slag content, as shown in Figure 6a,b. The range of 3450~3700 cm^−1^ is related to the stretching vibration band of H−OH and the bending vibration of the H−OH bond at 1640~1650 cm^−1^, which can be attributed to the presence of bound water present in the alkali-activated products [36,37].

The peak at 992 cm^−1^ represents the stretching vibration of Si-O of the SiO_4_ tetrahedron, indicating the formation of C-A-S-H gel [38]. Moreover, the asymmetric stretching vibration of T-O-Si (T = Si or Al) in the range of 1300~800 cm^−1^ corresponds to the structural units of silicon-oxygen tetrahedron ([SiO_4_]^4−^) and aluminum-oxygen tetrahedron ([AlO_4_]^5−^) in N-A-S-H gel phase [39]. Under the same sealed-curing conditions, the peak of C-S-H gel changes sharply with the increase in the slag content. Figure 6c shows the FT-IR spectrum of B13 (i.e., *W_SG_* = 50%, *M* = 1.4, *R_A_* = 50%). The peak of T-O-Si shifted to the low wave number (1004 cm^−1^ to 993 cm^−1^), indicating that although AA-FASM under different curing conditions had the same reaction products, the sealing condition was more favorable for sufficient reaction.

#### 3.1.6. Pore Structure

**Figure 7:**
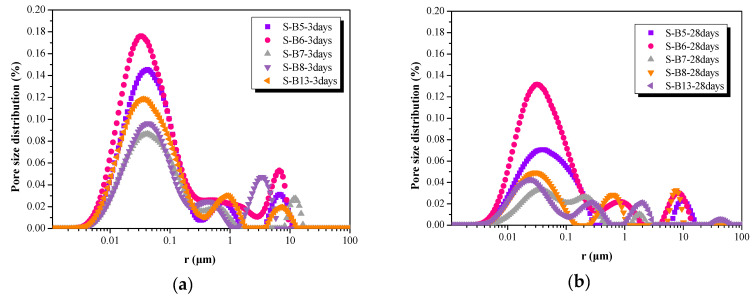
Pore size distribution of AA-FASM under seal-curing condition at (**a**) 3 days and (**b**) 28 days.

The pore size distribution curves of AA-FASM samples under the sealed-curing conditions are shown in Figure 7. At the curing age of 3 days, the pore size was mostly distributed in the range of 10 and 500 nm regardless of the content of fly ash (B5, B6) or slag (B7, B8 and B13). In contrast, the pore size of B7, B8 and B13 was mainly concentrated in the range of 10~100 nm at 28 days. Table 4 shows the porosity of AA-FASM at different ages adopted with sealed curing. It can be found that the B7 (*W_SG_* = 75%, *R_A_* = 45%) cured for 3 and 28 days exhibits the minimum porosity, which was 3.523% and 1.360%, respectively. Compared with the 3-day cured samples, the porosity of B5, B6, B7, B8 and B13 decreased by 47.67%, 27.29%, 61.40%, 51.25% and 65.14% at 28 days, respectively. In general, the reactivity of slag is higher than that of fly ash at room temperature. Due to the accumulation effect of particles and the coexistence of alkali-activated reaction products (C-S-H and N-A-S-H), the pore structure of the alkali-activated fly-ash–slag material is significantly refined and improved with the increase in curing age [40].

**Figure 8:**
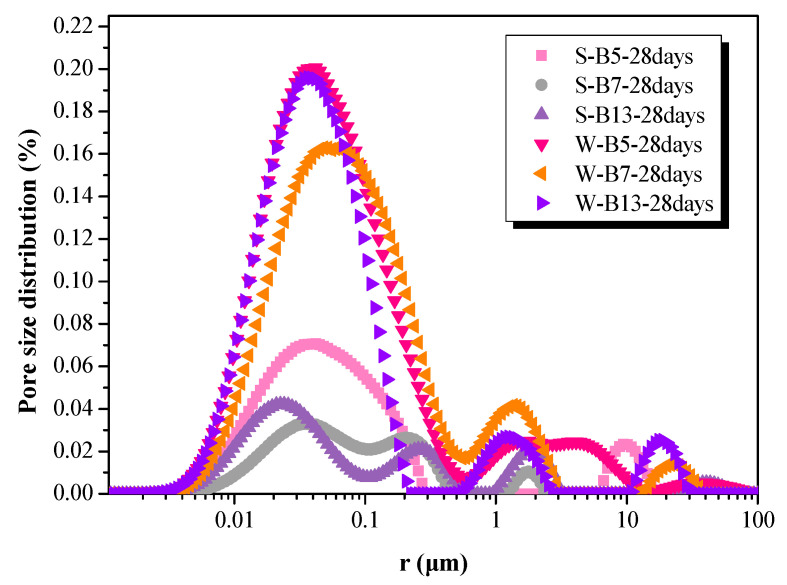
Pore size distribution of AA-FASM under seal- and water-saturation-curing conditions.

Figure 8 shows the comparison of the pore size distribution of AA-FASM under sealed- and water-saturation-curing conditions. At the age of 28 days, for the samples of S-B7 and S-B13 with *W_SG_* > 50% and *R_A_* ≤ 50%, the pore size gradually decreased due to the continuous formation of C-S-H gel and was mainly concentrated in the range of 10~100 nm. According to the reaction mechanism of geopolymerization, it can be known that the water-saturation curing with high humidity would hinder the reaction in the later period. The looser pore structure formed in the water-saturation-cured samples was large, and the pore size also varied from 10 nm to 500 nm.

**Figure 9:**
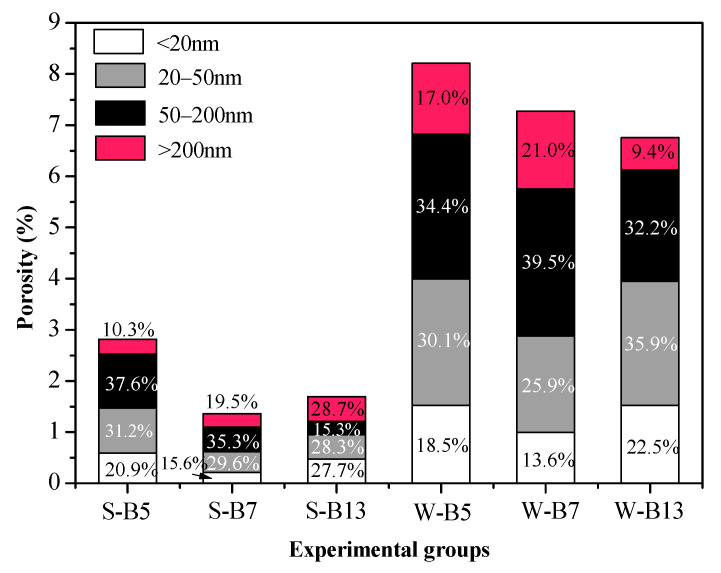
Porosity of AA–FASM under seal– and water–saturation–curing conditions.

The porosity of the S-B7 sample was approximately 5.35 times lower than the W-B7 (7.278%) sample cured for 28 days. Furthermore, the porosities of the S-B5 and S-B13 samples were also 2.92 and 3.99 times lower than the W-B5 and W-B13 samples, respectively (see Figure 9). The figure indicated that the pore structure of AA-FASM paste is mainly composed of small capillary pores, accompanied by a small amount of large capillary pores. The proportion of pores in the range of 0~200 nm accounts for more than 70% of the total porosity, and the pores in the range of 0–50 nm account for 39.5–58.4% of the total porosity. A decrease in the volume and size of pores and an increase in compressive strength are attributed to the addition of slag, decreased activator dosage and sealed-curing condition. However, water-saturation curing is not beneficial for geopolymerization and dry curing prone to initiate microcracks.

#### 3.1.7. BSE Morphology

**Figure 10:**
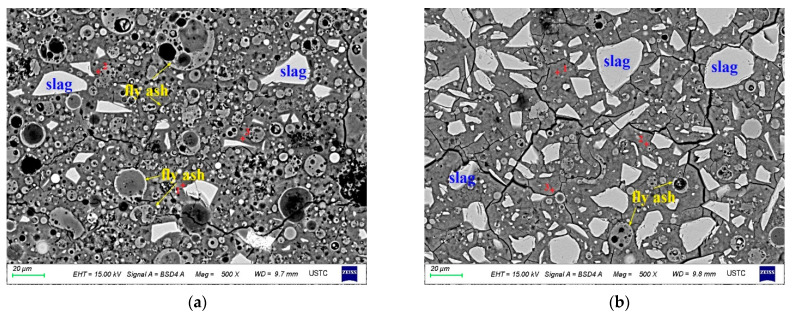
Backscattered morphology of (**a**) S-B5 and (**b**) S-B7.

S-B5 and S-B7 had a typical microstructure of AA-FASM, which was composed of the gel phase and unreacted fly ash and slag remnants embedded in the matrix (see Figure 10). There was a large number of unreacted fly ash particles (1–40 μm) in S-B5. The surface of fly ash remnants was corroded by alkali. A few of the fly ash spheres maintained a relatively complete particle morphology. For the S-B7 sample with 75% slag content, the gel phase contained more unreacted slag remnants and a smaller amount of fly ash remnants; compared with S-B5, its gel phase was more homogeneous. The main product formed in the sample with a high slag content is C-S-H or C-A-S-H type gel, thus macroscopic compressive strength is improved.

**Figure 11:**
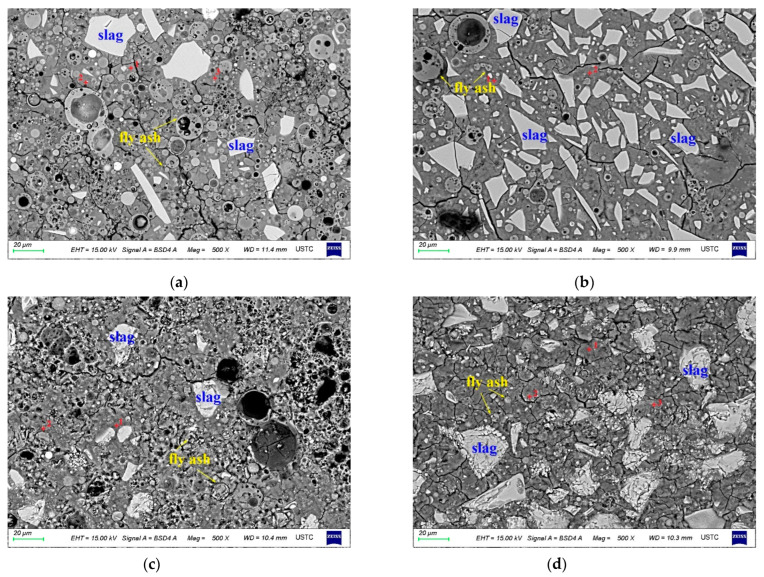
Backscattering morphology of B5 and B7 under dry- and water-saturation-curing conditions. (**a**) D-B5. (**b**) D-B7. (**c**) W-B5. (**d**) W-B7.

Figure 11 shows the BSE morphology of the B-5 and B-7 samples after 28 days of dry and water-saturation curing. Regardless of the change in curing conditions, the morphology of AA-FASM was similar, and many obvious unreacted remnants were present in the matrix. However, the difference was that the morphology of the specimen placed in the water was relatively loose. In addition to water-saturation curing, the boundaries between the unreacted remnants and the gel matrix were clear. Under the curing conditions with high humidity, microcracks developed in the gel phase and the residual particles. On the one hand, the microcracking development was related to the shrinkage of drying and to water loss using an oven. On the other hand, it may be due to the leaching of alkaline ions from the matrix, and the formation of pores due to leaching leads to the large porosity of the water-saturation-cured sample.

In the BSE images, the C-S-H and N-A-S-H gel phases cannot be clearly distinguished. By calculating the average Ca/Si ratios of the gel phase (see Table 5), it was found that with the increase in the slag content, the average Ca/Si exhibited an increasing trend up to 0.7581. The sequence of average Ca/Si ratios presented in the samples with different curing conditions was sealing > drying > water saturation, indicating that the isolation of external moisture exchange during curing can promote the formation of the gel phase. Higher humidity in water-saturation curing inhibits the polycondensation of silicate and aluminate monomers and hinders the formation of network-structured aluminosilicate gels [41]. For the sample with dry curing, the reduction in moisture causes cracking and weakens the participation of water in the alkali activation process [31]; as a consequence, compressive strength development is impaired. In contrast, sealed curing promotes the microstructure of AA-FASM products to be more homogeneous and compact.

Based on the results of AA-FASM under the sealed-curing condition, the response surface models of the compressive strength of AA-FASM under the interaction factors are also established, and the mechanism behind this model is further discussed. The purpose of establishing a model is to predict performance change trends such as compressive strength and choose an appropriate parameter range.

### 3.2. Establishment of Response Surface

#### 3.2.1. Test Results

Table 6 shows the comparison of compressive strength, cracks, initial setting and final setting time of 17 groups of sealed-curing samples. No cracks (N), fewer cracks (L) and more cracks (M) were used to describe the numbers of microcracks. The microcracks in AA-FASM samples were remarkedly influenced by *W_SG_*, *M* and *R_A_*. For instance, when the slag content was 75%, the shortest initial and final setting time was 50 min and 74 min, respectively. However, the longest initial and final setting time occurred, which was 317 min and 416 min, respectively, at the ratio of 25% slag.

#### 3.2.2. Second-Order Response Model

A mathematical model is established to explore the influence of interaction on strength development [42,43], as shown in Formula (4).

(4) $$Y=\beta_{0}+\sum_{i=1}^{k}\beta_{i}\chi_{i}+\sum_{i≺j}^{k}\beta_{ij}\chi_{i}\chi_{j}+\sum_{i=1}^{k}\beta_{ii}\chi_{i}{}^{2}+\varepsilon$$

where *Y* is the predicted value; *β_0_*, *β_i_*, *β_ii_* and *β_ij_* are constant and regression coefficients, respectively; *χ_i_* and *χ_j_* are variable factors; and *ε* is the systematic error. The second-order response surface models of compressive strength *R* and *W_SG_* (represented by *A*), *M* (represented by *B*) and *R_A_* (represented by *C*) are established as follows:

$$\begin{array}{l} R_{1}=383.25538+2.30118\times A+14.08375\times B-16.66\times C-0.31325\times A\times B-0.017280\times A\times C\\ -0.19125\times B\times C-5.7568\times 10^{-3}\times A^{2}+2.18437\times B^{2}+0.17128\times C^{2} \end{array}$$

$$\begin{array}{l} R_{3}=126.35176+1.2629\times A+46.42925\times B-5.8466\times C+0.0985\times A\times B-6.36\times 10^{-3}\times A\times C\\ -0.94875\times B\times C-6.62296\times 10^{-3}\times A^{2}-2.10531\times B^{2}+0.066726\times C^{2} \end{array}$$

$$\begin{array}{l} R_{28}=114.18325+1.26751\times A+26.07063\times B-3.28125\times C-0.017\times A\times B-0.02154\times A\times C\\ +0.57375\times B\times C-1.2296\times 10^{-3}\times A^{2}-19.99063\times B^{2}+0.02136\times C^{2} \end{array}$$

#### 3.2.3. Significance Test

The variance analysis of the second-order response surface model is given in Table 7, and the correlation coefficient *R*^2^ and *p*-Value are used to analyze the significance of the response model of compressive strength.

The results show that the *R*^2^ of the strength models at three curing ages was 0.9882, 0.9942 and 0.9659, and the *p*-Value of models was less than 0.05, indicating that the response surface model was significant and could be applied to the prediction of the compressive strength of AA-FASM paste.

#### 3.2.4. Response Surface

(1) Interaction of W_SG_/M

**Figure 12:**
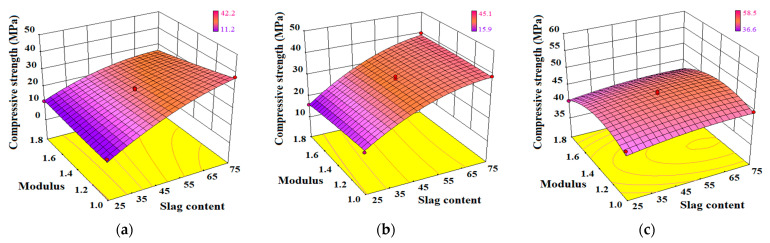
Effect of interaction between *W_SG_* and *M* on the compressive strength model of AA-FASM. (**a**) 1 day. (**b**) 3 days. (**c**) 28 days.

The effect of the interaction between the slag content *W_SG_* and activator modulus *M* on the compressive strength model of AA-FASM is shown in Figure 12. From 1 to 28 days, the response surface of compressive strength exhibited three stages of change: protruding corner, rising arc-shaped and upward convex surface. The early strength was not affected by *M* (when *W_SG_* ≤ 50%), and the basic trend of a rising arc-shaped surface at 3 days did not change with the increase in *W_SG_* and *M*. At the early 1-day and 3-day curing ages, the increase in slag content significantly increased the CaO/(SiO_2_+Al_2_O_3_) ratio from 0.13 to 0.44, regardless of the modulus of 1.0,1.4 or 1.8, which led to the rise of the surface. At 28 days, no matter the amount of slag, the strength surface was convex. As shown in Figure 12c, the CaO/(SiO_2_+Al_2_O_3_) ratios of the four endpoints at the corners of the surface were 0.131, 0.136, 0.418 and 0.436, respectively. Although the ratio difference between the endpoints was 0.3, the compressive strength of the four endpoints was lower than that of the center point. It also indicated that a high or low modulus will hinder the development of geopolymer strength. In addition, similar to the AA-FASM, the activated slag system, explored by Gao et al. [43], is prominently influenced by the activator modulus.

The shape change of the response surface may be related to Ca^2+^ content in the alkali-activated system [12] and the activity of the water glass activator [44]. The increased slag content promotes the increase in Ca^2+^ concentration during alkali dissolution, where C-S-H acted as a nucleation site for the formation of N-A-S-H [35]. In addition, water glass (sodium silicate) with a high modulus has low reactivity due to the characteristics of high viscosity, a low number of monopolymers and a high polymerization degree of silicon-oxygen tetrahedron, while the activator with a low modulus presents an opposite trend [44]. The report [31] pointed out that when the modulus is *M* = 3.0, the polymerized structural units of [SiO_4_]^4-^ in water glass are mostly Q^3^ (layered or chain), Q^2^ (chain) and Q^1^ (dimer), when *M* = 1.5, Q^0^ (monomer) increases and Q^3^ decreases in quantity, and its activity increases. Too-high and too-low moduli are not good for geopolymerization.

(2) Interaction of W_SG_/R_A_

**Figure 13:**
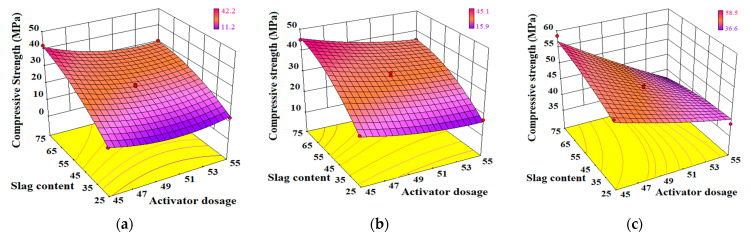
Effect of interaction between *W_SG_* and *R_A_* on the compressive strength model of AA-FASM. (**a**) 1 day. (**b**) 3 days. (**c**) 28 days.

The effect of the interaction between the slag content *W_SG_* and activator dosage *R_A_* on the compressive strength model of AA-FASM is shown in Figure 13. The compressive strength surfaces from 1 to 28 days show similar slope growth, but at 28 days, there is a significant difference of about 37% between the predicted strengths of *R_A_* = 45% and *R_A_* = 55% (*W_SG_* = 75%). Under the same slag content, specimens with low CaO/(SiO_2_+Al_2_O_3_) in the alkali-activated system tended to have a low compressive strength distribution, which can also be reflected by the size of the alkaline solution content, such as B8 less than B7 strength with CaO/(SiO_2_+Al_2_O_3_) of 0.432.

The influence of *R_A_* in the early ages was not obvious, which was similar to the literature [45]. The interaction between *W_SG_* and *R_A_* promoted the compressive strength of AA-FASM to increase with the increase in slag content and the decrease in activator dosage, and this phenomenon is also reflected in Figure 2. The slope growth is mainly related to the degree of slag and fly ash activated by the alkaline solution [44]. In this experiment, *R_A_* in the range of 45~55% did not cause an adverse effect due to excessive alkalinity. Furthermore, for an alkali activator with a high dosage, there is a relatively large amount of water, and an increase in water content will be detrimental to the geopolymerization [41]. Similarly, there was also an increase in mass loss (Figure 3a) and linear shrinkage (Figure 4a) caused by the high dosage of the activator. Although the interaction between *W_SG_* and *R_A_* does not cause complex changes in the response surface of compressive strength, it still shows obvious trend changes.

(3) Interaction of M/R_A_

**Figure 14:**
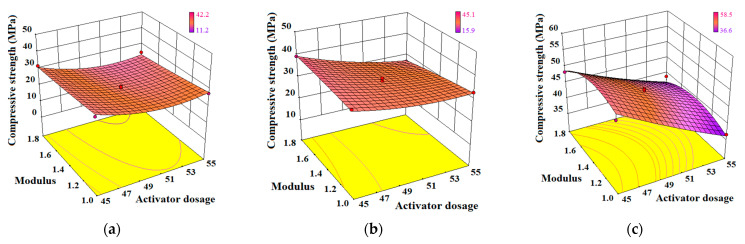
Effect of interaction between *M* and *R_A_* on the compressive strength model of AA-FASM. (**a**) 1 day. (**b**) 3 days. (**c**) 28 days.

The effect of the interaction between the activator modulus *M* and activator dosage *R_A_* on the compressive strength model of AA-FASM is shown in Figure 14. Due to the disadvantage of the high alkalinity of the alkali-activated reaction and the relatively high proportion of water in a large activator dosage, the strength model under the interaction changes from the gentle concave after 1-day curing to an inclined convex surface after 28-day curing. Compared with *W_SG_*/*M* and *W_SG_*/*R_A_*, the differences between the predicted values of compressive strength under the interaction of *M*/*R_A_* were relatively small. The good adaptability of the response surface was verified by the analysis of variance or the evolution of surface shape, which was helpful for the selection of parameters for the preparation of AA-FASM.

#### 3.2.5. Selection of Parameter

**Figure 15:**
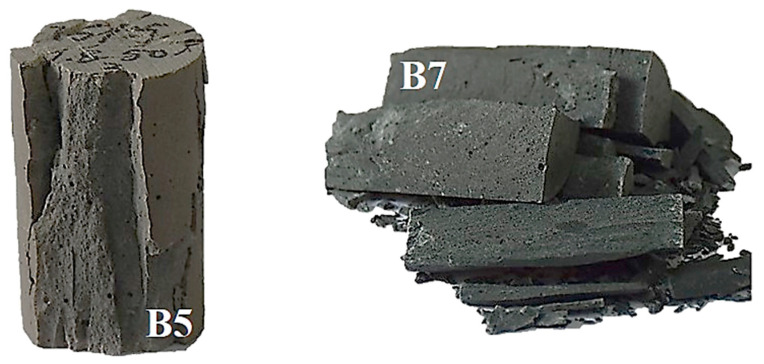
Comparison of the fracture state of B5 and B7 of AA-FASM after compressive strength.

Figure 15 shows the fracture states of B5 (75% fly ash) and B7 (75% slag) of AA-FASM after the compressive strength test. The brittleness and flaky structure were associated with a high slag content; in contrast, the high content of the fly ash sample was relatively complete. When the AA-FASM with a high slag content was compressed, a sudden fracture appeared when the peak load was reached. Therefore, in the selection of mix parameters, the maximum strength should not be pursued alone, but the damage characteristics should also be considered to improve the safety of engineering applications.

**Figure 16:**
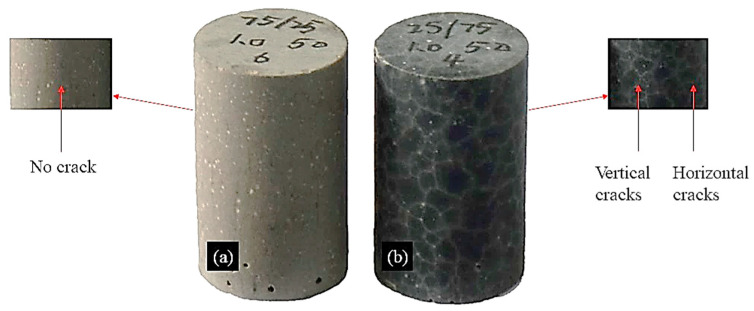
Comparison of cracks between B1 (**a**) and B3 (**b**) of AA-FASM.

Figure 16 shows the comparison of the cracks between B1 and B3. Compared with B1 (*W_SG_* = 25%) without cracks, a denser curve or arc crack network appeared on the surface of B3 (*W_SG_* = 75%). The presence of microcracks indicated that it has great shrinkage sensitivity. It is generally believed that the hardened paste of the alkali-activated material is mainly composed of gels [36,46], which give it higher compressive strength but also higher shrinkage. Reference [47] also points out that the large shrinkage of sodium silicate-activated slag is associated with the dehydration of silica gel with a high water content or silica-rich gel products.

Combining the basic mechanical properties, microstructure and response surface models and cracking characteristics of AA-FASM, it was recommended that the optimal proportioning parameters and curing condition be selected as follows: slag content *W_SG_* = 50%, activator modulus *M* = 1.4, activator dosage *R_A_* = 50% and the seal-curing condition.

The high-correlation regression model showed that the response surface method can be used to predict the compressive strength of mixtures, which was similar to the results of the studies [43,48,49]. Other researchers have also evaluated the possibility of alkali-activated materials prepared by using the response surface analysis method for backfill, sidewalk slabs and the rapid repair of backfill and runways [43,48] and demonstrated their feasibility. It should be noted that the model in this work cannot be used as a unified model of alkali-activated materials, and the mechanical properties of the final product depend on the properties of raw materials in different regions. However, the related model is still an effective method to optimize the final product properties of alkali-activated materials. This research contributes to the production of sustainable alkali-activated concrete using locally available materials and provides data support for the future development of integrated prediction models.

## 4. Conclusions

In this paper, the effects of seal, dry and water-saturation curing on the performance and microstructure of alkali-activated fly-ash–slag materials were studied. Meanwhile, the interactions among the factors of slag content, activator modulus and dosage were verified by using the response surface model. The main conclusions of this study can be drawn as follows:

(1) The compressive strength is up to 59 MPa after 28 days of sealed curing, while the strengths of the group with the same mix proportion under drying and water-saturation curing decrease by 9.8% and 13.7%, respectively. Sealed curing locks the water within the system, so the sample has the smallest mass loss, linear shrinkage, total porosity and refined pore size.
(2) The correlation coefficients of the response surface models for the AA-FASMs cured for 1 day, 3 days and 28 days are both higher than 0.95, and the *p*-values are all less than 0.05. These results show that compressive strength can be predicted precisely using the response surface method.
(3) The slag content *W_SG_*, activator modulus *M* and activator dosage *R_A_* have significant effects on the strength development of AA-FASM. Because of the interaction of *W_SG_*/*M*, the response surface experiences a change in shape from a protruding corner, rising arc-shaped to convex surface. The response surface under the interaction of *W_SG_*/*R_A_* is mainly characterized by slope growth, while the interaction surface of *M*/*R_A_* shows a transition from a concave to a convex shape.
(4) In the studied range of factors, the optimal mix proportion parameter and curing conditions of AA-FASM are slag content of 50%, activator modulus of 1.4, activator dosage of 50% and sealed curing.

## Figures and Tables

**Table 1:** Oxide composition and loss on ignition of raw materials (weight in %).

Materials	SiO_2_	Al_2_O_3_	CaO	Fe_2_O_3_	MgO	LOI ^a^
Fly ash	56.15	32.42	3.18	1.00	0.25	0.88
Slag	32.58	15.04	38.25	0.30	8.57	0

^a^ Note: LOI represents the loss on ignition.

**Table 2:** Box–Behnken design with three factors and three levels.

Factor	Code	Level		
		−1	0	1
*W_SG_* (%)	X_1_	25	50	75
*M*	X_2_	1.0	1.4	1.8
*R_A_* (%)	X_3_	45	50	55

**Table 3:** Design scheme of response surface analysis under seal-curing condition.

Group	*W_SG_* (%)	*M*	*R_A_* (%)	*CaO/(SiO_2_+Al_2_O_3_)* *^∗^*
B1	25	1.0	50	0.136
B2	25	1.8	50	0.131
B3	75	1.0	50	0.436
B4	75	1.8	50	0.418
B5	25	1.4	45	0.135
B6	25	1.4	55	0.131
B7	75	1.4	45	0.432
B8	75	1.4	55	0.418
B9	50	1.0	45	0.269
B10	50	1.0	55	0.262
B11	50	1.8	45	0.260
B12	50	1.8	55	0.253
B13	50	1.4	50	0.261
B14	50	1.4	50	0.261
B15	50	1.4	50	0.261
B16	50	1.4	50	0.261
B17	50	1.4	50	0.261

*Note:* *^∗^ mass ratio.*

**Table 4:** Porosity of AA-FASM under seal-curing condition (%).

Days	S-B5	S-B6	S-B7	S-B8	S-B13
3	5.377	7.04	3.523	3.975	4.862
28	2.814	5.119	1.360	1.938	1.695

**Table 5:** Average Ca/Si ratio of geopolymer by EDS analysis.

	S-B5	S-B7	D-B5	D-B7	W-B5	W-B7
Ca/Si	0.4342	0.7581	0.4145	0.6664	0.4098	0.6120

**Table 6:** Test results of AA-FASM.

Numbers	Compressive Strength (MPa)			Cracks			Setting Time (min)	
	1d	3d	28d	1d	3d	28d	Initial	Final
B1	11.2	18.8	43.1	N	N	N	317	416
B2	11.4	15.9	40.3	N	N	N	131	160
B3	37.6	38.9	44.6	N	L	M	101	142
B4	25.3	40.0	41.2	N	L	M	50	74
B5	15.7	23.5	50.0	N	N	L	217	280
B6	12.8	17.3	38.9	N	N	L	288	353
B7	42.2	45.1	58.5	N	L	M	55	76
B8	30.6	35.7	36.7	N	L	M	73	101
B9	32.9	37.8	50.8	N	L	L	117	164
B10	27.6	32.9	36.6	N	L	L	157	236
B11	31.7	39.3	48.3	N	N	L	63	83
B12	24.9	26.9	38.7	N	N	L	74	92
B13	24.6	32.7	46.3	N	L	L	101	141
B14	24.5	32.3	46.9	N	L	L	91	139
B15	25.2	33.1	46.6	N	L	L	98	144
B16	23.2	34.3	46.2	N	L	L	89	142
B17	25.6	32.1	45.5	N	L	L	86	137

**Table 7:** Analysis of variance of response surface model.

Source	1d		3d		28d	
	F Value	*p*-Value	F Value	*p*-Value	F Value	*p*-Value
Model	64.89	<0.0001	134.17	<0.0001	22.04	0.0002
X_1_-X_1_	435.57	<0.0001	947.73	<0.0001	3.80	0.0922
X_2_-X_2_	15.74	0.0054	5.37	0.0536	2.17	0.1843
X_3_-X_3_	42.91	0.0003	144.25	<0.0001	159.97	<0.0001
X_1 × 2_	19.11	0.0033	4.16	0.0808	0.0046	0.8364
X_1_X_3_	9.09	0.0195	2.71	0.1437	11.52	0.0115
X_2_X_3_	0.28	0.6100	15.44	0.0057	2.09	0.1913
X_1_^2^	26.54	0.0013	77.34	<0.0001	0.99	0.3534
X_2_^2^	0.25	0.6321	0.51	0.4974	17.11	0.0044
X_3_^2^	37.60	0.0005	12.56	0.0094	0.48	0.5121
Lack of Fit	4.78	0.0824	1.64	0.3153	18.43	0.0083
R^2^	0.9882		0.9942		0.9659	

Note: *p*-value ≤ 0.01 is highly significant; *p*-value ≤ 0.05 is significant; *p*-value > 0.05 is not significant.

## Data Availability

Not applicable.
